# Franklin Medical College of Philadelphia

**Published:** 1846-05

**Authors:** 


### FRANKLIN MEDICAL COLLEGE OF PHILADELPHIA.

Students who may repair to Philadelphia in the coming autumn will find there four medical schools between which to choose. The Franklin Medical College takes its place in that honored sisterhood, to do service in the cause of medical education, and compete for its share of pupils and renown. At first, the impression made upon the mind by the annunciation that a *fourth* school has been opened in Philadelphia, is, that our brethren in the city of brotherly love are over-doing the thing, and that some of these new enterprises must prove a failure. Such, for a long time, was the impression in respect to the Jefferson Medical College; but after years of hard toil, many changes of professors, and many reverses, it has at last fixed itself firmly in public confidence, and now vies in the number of its pupils with its ancient rival, the University. In the meantime a third school, the Pennsylvania Medical College, was set on foot, and seems to be enjoying a satisfactory amount of success. And still the *old* School, the University, is as prosperous as ever. For nearly twenty years before the Jefferson Medical College was chartered, the classes at the University amounted to more than four hundred; but very seldom were they larger than the class which rewarded the labors of the professors during the season which has just closed. The effect, therefore, of the multiplication of schools in Philadelphia has been to attract students in still increasing numbers to that city, and not to cripple those first established. So long as this continues to be the case there can be no objection to an increase. Beyond a certain number it is not desirable that students should congregate in rooms where much of the teaching must be demonstrative. When that number is exceeded, hearing becomes very uncertain, and seeing quite impossible.

But on the other hand, in the present rage for creating medical schools it is very probable that many will be started which cannot for a long time attract such classes as will make it an object with men of talents and learning to hold places in them. The effect of this upon the character of the profession is easily foreseen. As it is for the benefit of students that the rooms be not too much crowded, so is it equally essential to teachers that they have something more before them than empty benches. But empty benches must be the allotment of some, if the present rate of increase should continue.

We do not believe, however, that this will be the share of our friends of the Franklin Medical College. A number of them are already known to fame; some of them have enjoyed experience as teachers, and their success cannot be looked upon as doubtful. We subjoin a list of their names and professorships.

Paul Beck Goddard, M.D., Anatomy and Histology.

C. C. Van Wyck, M.D., Principles and Practice of Surgery.

Meredith Clymer, M.D., Principles and Practice of Medicine.

John Barclay Biddle, M.D., Materia Medica and Therapeutics.

David Hunter Tucker, M.D., Obstetrics and Diseases of Women and Children.

Levin S. Joynes, M.D., Physiology and Legal Medicine.

James B. Rogers, M.D., General and Organic Chemistry.

Demonstrator of Anatomy, Joseph Leidy, M.D.

